# Personal recovery of adolescents with mental health conditions in the community: empirically-based practical implications

**DOI:** 10.1186/s13584-025-00725-0

**Published:** 2025-10-30

**Authors:** Hila Tuaf, Hod Orkibi

**Affiliations:** Faculty of Social Welfare and Health Sciences, Drama & Health Science Lab, and the Emili Sagol Creative Arts Therapies Research Center, University of Haifa, Mount Carmel, Haifa, 31905 Israel

**Keywords:** Adolescents, Youth, Mental health, Recovery, Continuity of care, Social integration

## Abstract

**Background:**

Approximately one in seven 10 to 19-year-olds cope with mental health conditions globally, which amounts to 13% of the overall burden of disease within this specific age demographic, and the prevalence is expected to rise. Neglecting the management of mental health conditions during adolescence has repercussions into adulthood that adversely affect both physical and mental well-being, and constrain opportunities for leading fulfilling lives.

**Main body:**

This integrative article introduces a new empirically-based model entitled “Continuity of Community Reintegration” (the CCR model) that acknowledges the emotional, social, and functional aspects of the personal recovery process of adolescents by considering the essential care providers, i.e., the family, community-based programs, schools, and clinics or hospitals. This model is based on a research project including a scoping review and qualitative data collected from 86 stakeholders involved in *Amitim for Youth*, the first community-based program for adolescents with mental health conditions in Israel.

**Conclusion:**

The model underscores that continuity of care across all the care providers is essential to optimize the personal recovery of these adolescents while emphasizing their agency, encouraging them to engage actively in decision-making, goal setting, and while guiding them towards reintegration into the community and personal recovery.

**Supplementary Information:**

The online version contains supplementary material available at 10.1186/s13584-025-00725-0.

## Background

The phase of adolescence is manifested by a surfeit of physiological, psychological, and social transformations that generate various challenges [1, 2]. As suggested in Erikson’s theory of psychosocial development [3], youth in this stage face the contrast of “identity versus role confusion” by engaging in the pursuit of personal identity and a quest for independence. Adolescents examine their social roles, values, beliefs, and goals, while distancing from parental influences. For adolescents with mental health conditions (MHC), this developmental task becomes particularly intricate. Such complexity can manifest as obstacles in the formation of a stable identity or, at times, the development of a negative identity [2].

“Mental health conditions” refers to an umbrella term encompassing a spectrum of psychiatric diagnoses. In adolescents, depression, anxiety, and behavioral disorders such as attention deficit hyperactivity disorder (ADHD) and conduct disorder are particularly prevalent, and contribute significantly to illness and disability [4]. In addition, eating disorders such as anorexia nervosa and bulimia nervosa, psychosis, suicide, self-harm, as well as risk-taking behaviors such as substance use or engaging in risky sexual behavior all fall within this comprehensive term.

On a global scale, approximately one out of every seven 10 to 19-year-old cope with MHC, which accounts for 13% of the overall burden of disease within this specific age demographic [4]. Suicide ranks as the fourth most prevalent cause of mortality in adolescents and young adults aged 15–29. MHC account for 16% of the global morbidity among adolescents [4], and is a major cause of mortality among adolescents in Europe [5]. A worldwide meta-analysis reported that approximately 13.4% of all children and adolescents cope with MHC [6] and the prevalence is expected to rise [7, 8]. Neglecting the management of MHC during adolescence has repercussions in adulthood, because it adversely affects these individuals’ physical and mental well-being and constrains opportunities for leading fulfilling lives later on [4].

## The treatment gap

Apart from the typical challenges associated with adolescence [2], those coping with MHC face heightened vulnerability to social exclusion, discrimination, and stigma. These risk factors not only inhibit their willingness to seek support [4] but also significantly account for the treatment gap [9, 10]. Findings from systematic review underscored that a substantial 92% of all adolescents with MHC perceive various social factors, including public stigma and embarrassment, as substantial barriers that hinder help-seeking behavior [11].

The mental health literature makes a crucial distinction between two types of stigma. Public stigma is characterized by detrimental labeling, prejudice, stereotypes and discrimination to isolate a group [12]. Self-stigma (or internalized stigma) is marked by the internal absorption of societal stigma impacting individuals’ self-perception [12]. Given these considerations, service providers must acknowledge and address the unique needs of adolescents with MHC throughout the recovery process by tailoring services with sensitivity and professionalism. Nevertheless, despite the high prevalence of MHC in children and adolescents, treatment rates remain insufficient, and point to the significant gap in treatment [11]. The COVID-19 pandemic exacerbated MHC in adolescents, thus stressing the need for long-term strategies and the greater accessibility of mental health services [13–15].

### The personal recovery approach: evolution of mental health approaches

The ‘medical model’ that dominated Western mental health systems for decades framed individuals with MHC as patients requiring extended hospitalization [16]. Recently, a paradigm shift has emerged. Over the past few decades, the personal recovery approach has gained traction, influencing rehabilitation policies in mental health settings [17]. Grounded in the principle of person-centered care, this approach prioritizes enhancing the quality of life for individuals with MHC, regardless of symptom severity. It underscores the facilitation of community integration, along with efforts to reinstate a sense of control, choice, independence, autonomy, responsibility, meaning, and hope [16, 17]. The term ‘clinical rehabilitation’ refers to an objective characterization, where the individual undergoes an evaluation by a trained professional, and which is primarily centered on outcomes such as symptom reduction. By contrast, ‘personal recovery’ relates to the subjective self-perception of individuals during their own recovery journey [18, 19].

The personal recovery approach is implemented in services tailored for adolescents that are attuned to their developmental needs for independence, self-efficacy, and self-determination [20–22]. Thus positioned as active consumers of services, these adolescents are encouraged to assert agency in their recovery journey, by actively participating in decision-making processes, and articulating their opinions and needs regarding the services provided [20, 23, 24]. Empirical assessments of programs tailored for adolescents have revealed heightened user satisfaction, in particular in the case of service teams characterized as friendly, respectful, supportive, and non-judgmental [25]. A survey participated by mental health professionals (MHP) with extensive experience in Australian Child and Adolescent Mental Health Services revealed that personal recovery for young people with MHC requires incorporating family and support networks in goal setting and decision-making, while ensuring that young people’s needs remain central [26].

## Continuity of care

Continuity of care is a medical term that refers to the longitudinal, consistent relationship between a physician and a patient [27, 28]. In optimal continuity of care, consumers feel a sense of affiliation with their care provider [28, 29]. Coordination between different services is particularly crucial for children and adolescents since they go through multiple transitions and are thus more exposed to the risks of discontinuity, which often results in fragmented care and disengagement from services [30]. A systematic review of mental health interventions in community and schools with an emphasis on the collaboration of different stakeholders (e.g., adolescents, family, teachers, MHP and community members) pointed to beneficial outcomes including a reduction in internalizing and externalizing symptoms, the promotion of personal well-being, as well as enhanced factors such as social support and engagement [31], although the specific impact of collaboration and best practice recommendations that can be drawn from these interventions remain unclear [31].

## Social integration

Social integration in adolescents pertains to the dynamic process where individuals forge connections, establish relationships, and participate in social activities within both peer circles and broader social networks. This concept encompasses the extent to which adolescents experience acceptance, inclusion, and involvement across diverse social contexts, such as in the family, school, and community. Social integration is a multifaceted construct shaped by numerous factors including social support, network size, network cohesion, perceived social relationships, social skills, and adherence to cultural norms [32–34]. Research has shown that social integration is fundamental to adolescents’ mental health, well-being, and adjustment [35–37]. Studies indicate that whether through face-to-face interactions or online platforms, social integration plays an essential role in shaping well-being of adolescents [35, 38].

To address the social integration of adolescents with MHC, it is important to consider the impact of various factors on their mental well-being. The COVID-19 pandemic exacerbated MHC among adolescents, and adolescents with pre-existing MHC were particularly vulnerable to isolation, the absence of routine, and limited access to health services [39]. Today, social media have become adolescents’ most popular leisure interest. While many adolescents acknowledge the positive side of social media, current research has found significant positive correlation between usage of social media and depression, anxiety, stress and aggression [40]. Robust social networks and positive interpersonal connections during adolescence have consistently been associated with enhanced psychosocial well-being and diminished levels of dysfunction [41, 42]. Social isolation and restricted social integration have been linked to greater depressive symptoms and additional challenges in mental health [43].

### **The Case of*Amitim for Youth***.

In 2001, the *Amitim* program for adults in Israel was established in adherence to the legislation (“Rehabilitation in the Community of Persons with Mental Disabilities Law of 2000”), aiming to include adults aged 18 and older, with a minimum 40% mental disability, who are eligible to the rehabilitation basket, as determined by the National Insurance Institute. With a collaborative effort between the Ministry of Health and the Israel Association of Community Centers, the *Amitim* program currently operates in 138 community centers in 79 locations in Israel, catering to over 5500 adults with MHC [44].

In January 2018, the Israel Association of Community Centers, in collaboration with the Ministry of Health, the Ministry of Education, and the Special Projects Fund of the National Insurance Institute, introduced the groundbreaking *Amitim for Youth* program. An inter-ministerial steering committee, comprising members from each stakeholder, played a pivotal role in the program’s establishment and initial implementation. Addressing the absence of community-based psychosocial rehabilitation services during adolescents’ leisure (after-school) hours, the program serves adolescents aged 12–18 with MHC.

The program operates today in 6 cities in Israel near psychiatric hospitals with youth departments and special education schools for children with MHC. Each district appoints a coordinator offering services to around 20 adolescents in local community centers. Participants receive an annual stipend of 1,200 Israeli shekels for engaging in community-based leisure activities. The goals of *Amitim for Youth* span three dimensions: youth, their families, and the broader community. For the youth, the program aims to promote socialization, a sense of community belonging, the ability to manage self-stigma, prevent repeated hospitalizations, achieve age-appropriate functioning aligned with personal goals, and find meaningful activities leading to satisfaction and self-actualization. For families, the program provides support through the coordinator, while for the wider community, the objective is to shift attitudes toward youth with MHC by fostering awareness [44, 45].

## Main text

### The continuity of community reintegration model for adolescents with MHC

The following section presents, for the first time, the empirically based “Continuity of Community Reintegration” model (CCR model) for the reintegration to the community of adolescents with MHC, grounded in our empirical findings [46, 47]. Our previous publications present a scoping review and practical implications of community-based programs for youth with MHC [46]; and the case study of *Amitim for Youth*, utilizing qualitative data (in-depth semi-structured interviews, focus groups, and the program’s steering committee) from 86 diverse stakeholders. The analysis revealed three core themes: the promotion of adolescent agency and independence; the cultivation of positive identity and meaning through creative activities; and the critical role of mentorship in fostering a sense of community belonging [47].

**Fig. 1:**
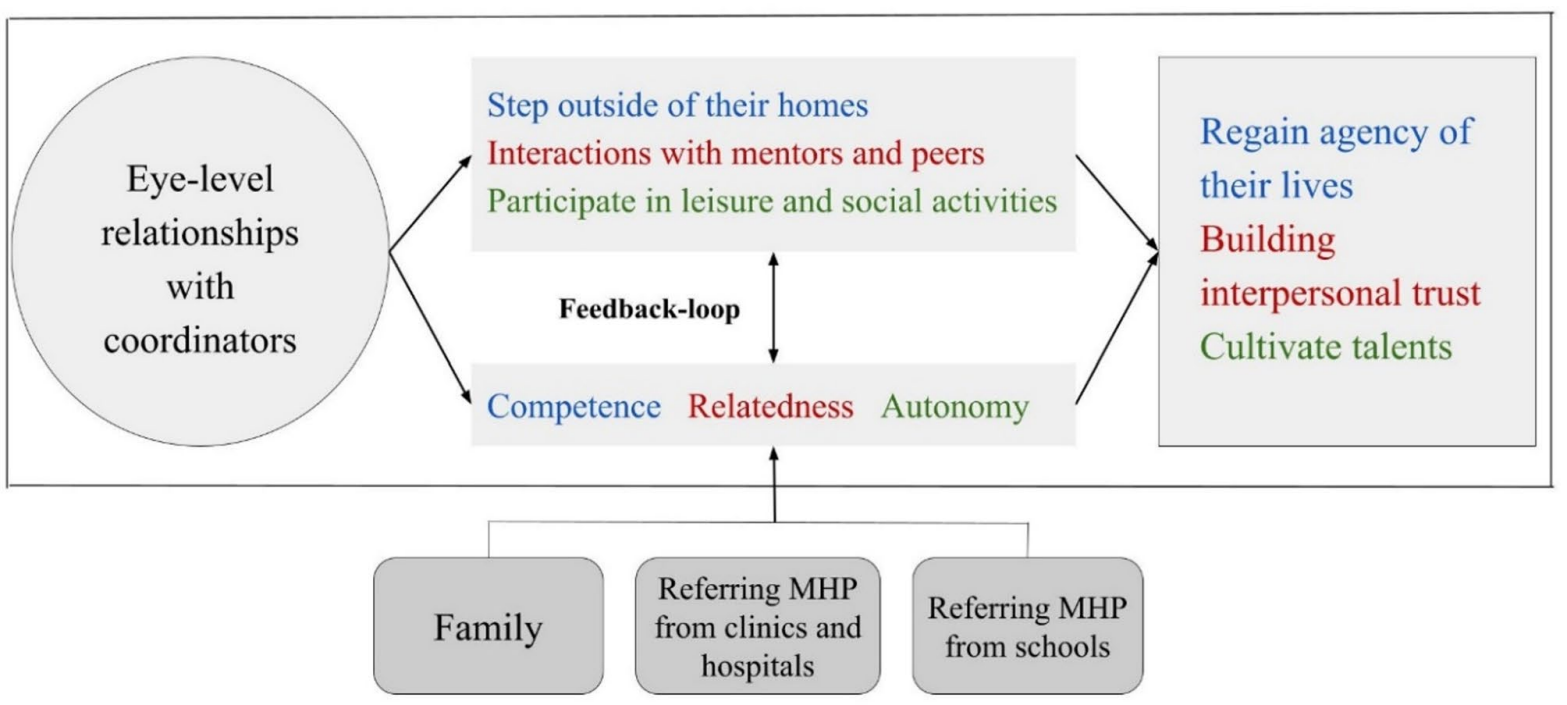
Continuity of community reintegration model (CCR). Note: This figure illustrates adolescents’ reintegration process in the specific community-based program and in other settings, with the support of essential care providers, thus echoing the feedback loop

The CCR model acknowledges the social, emotional, and functional aspects of adolescents’ personal recovery process. As can be seen in Fig. 1, the reintegration journey begins with the prerequisite that the coordinators should form an eye-level relationship with these adolescents. After this initial step, two parallel pathways evolve and develop as the relationship deepens. In the practical pathway, adolescents gradually leave their homes to attend individual meetings with coordinators to establish personal goals, identify their preferred leisure activities, and later engage with mentors and peers in various leisure and social activities. Simultaneously, in the emotional pathway, the adolescents experience the continued development of competence, relatedness, and autonomy. This is consistent with the three fundamental psychological needs of Self-Determination Theory: competence, relatedness, and autonomy [48]. The reciprocal nature of these interconnected pathways forms a feedback loop, where each reinforces the other, leading to the empowerment of these adolescents to reclaim agency in their lives, establish interpersonal trust with their surroundings, and nurture their talents. Ultimately, these pathways contribute to the fostering of personal recovery, development, and reintegration of these adolescents into the community.

## Best practice recommendations for implementation and operation of CCR

**Continuity of care**. It is recommended that the collaboration of all essential care providers (family, community-based programs, and MHP in clinics/hospitals and schools) begins at the start of the program with a meeting to assess the adolescent’s baseline level of independence and community integration. This evaluation then serves as a foundation for determining the support needed during the community-based program (frequency of sessions with the coordinator, mentoring, activities, etc.), and in other settings such as clinic/hospital and school. To finalize the personalized tailored working plan for the adolescents’ recovery process, the adolescents’ needs, but also their personal goals and decisions should be addressed. A minimum of two follow-up meetings with all care providers are recommended at key points during the program year (midpoint and end). The purpose of these meetings is to discuss the adolescents’ practical and emotional pathways while addressing their competence, relatedness and autonomy. In addition, to sustain continuity of care and optimize the adolescents’ personal recovery, care providers must be in continuous communication, and share information on the adolescents’ progress towards community reintegration.

Special emphasis should be given to clarifying and defining the roles of each service provider working with the adolescents, as a function of their training, experience and supervision. Nevertheless, there is a thin line between recovery and therapy that should be discussed, and involves setting boundaries to assist the coordinators, MHP and school staff in providing more comprehensive support to these adolescents. Ensuring the goals of recovery (pursued by coordinator) and therapy (pursued by therapist) complete each other, while the therapist oversees the processing of emotional experiences, and the coordinator is responsible for the functional aspects of recovery (e.g., leaving the home environment to engage in community interactions).

All essential care providers (family and MHP) must strengthen the adolescents’ reintegration by encouraging them to continue the process by offering additional opportunities where the adolescents can practice the feedback loop, and strengthen competence, relatedness and autonomy. Family and parents can empower the adolescents’ competence by encouraging them to engage in daily activities such as grocery shopping or meal preparation. Providing opportunities for decision-making, such as selecting a family movie or engaging in joint activities and games of their choice, can contribute to enhancing relatedness and autonomy. It is also recommended that the school counselor initiate informal close social interactions with peers with common interests as adolescents to reinforce relatedness and social integration. The adolescents’ teacher should oversee daily functioning, by providing opportunities to carry out small tasks to increase competence, relatedness, and autonomy, depending on the adolescents’ condition.

**Designing the program consistent with the personal recovery approach**. It is recommended that program goals adhere to the personal recovery approach, by encouraging the adolescents to take agency over their own recovery process through decision-making and proactive engagement, according to the consumers (adolescents) and stakeholders of the specific community-based program [47], and as aligned with programs worldwide [46]. Using inclusive terminology in both the program name and description are also imperative for various reasons and fundamental principles of the recovery approach.

Specifically, it is recommended that in line with the recovery approach, the terms “recovery” and “coping” will be utilized in these programs since they are more suitable than the term “rehabilitation”. As a member of the steering committee argued, discussing rehabilitation in adolescence is improper because it typically reflects an extended history of coping with MHC, which does not correspond to adolescence (see quote 1 in Online Supplements).

Second, it is recommended that programs be normalized by being inclusive, to enable adolescents with MHC without a formal psychiatric diagnosis to participate. This is consistent with previous research indicating that adolescents may be hesitant to seek professional help out of fear of peer stigmatization [11, 49, 50]. This step will help minimize the fear of labeling and stigma among consumers and the community and provide a comprehensive response to all adolescents with MHC, regardless of whether they have a formal diagnosis. In particular, programs should be implemented as a strategy to prevent further deterioration and hospitalization, thus enabling adolescents to reintegrate into the community. Most of the programs presented in our scoping review [46] defined their intended audience as “adolescents” and/or “young adults” with MHC but refrained from explicitly delineating the specific psychiatric conditions of the users. This deliberate choice of broad terminology appeared strategic, and was aimed at sidestepping stigma and labeling to engage with a more extensive demographic.

Third, it is recommended that program providers consider adolescents’ basic psychological needs for competence, relatedness, and autonomy. Competence can be achieved by encouraging adolescents to overcome new challenges, e.g., using public transportation. Relatedness can be achieved by the establishment of relationships with coordinators and volunteer youth supporters (i.e., mentors) and by promoting interactions with peers in structured social activities. Autonomy can be achieved by encouraging adolescents to participate in leisure activities of their personal choice and enabling them to decrease dependence on their parents and experience independence by leaving the home environment to engage in community interaction and activities.

**Recruitment of coordinators for the program**. The findings revealed that a significant strength of the specific community-based program was its human capital, as concretized in the selection of coordinators with appropriate qualifications, experience, and personal qualities [47]. It is recommended that coordinators hold a master’s degree in the fields of rehabilitation, therapy, or social work, with a minimum of three years of work experience with adolescents in the mental health field. Coordinators should be mobile (either have their own car or be allocated one) and possess personal qualities such as good interpersonal skills, teamwork abilities, commitment, dedication, empathy, sensitivity, initiative, assertiveness, creativity, flexibility, a sense of professional competence and an ability to work independently. The coordinators should act as the representatives of the program and have presentation skills to enhance program promotion and collaborate with other professionals.

**Service accessibility and referral process**. It is recommended that policy makers aim to increase accessibility by a significant allocation of financial resources that will insure free or low-cost services for adolescents and their families [21, 23, 51]. The entry point to these services must be user-friendly, particularly because it constitutes the adolescents’ first interaction with the service. This need was stated clearly by the adolescents’ parents in the specific community-based program who reported great satisfaction with the referral process which they described as “easy, fast, and simple.” In most cases, the program was recommended to parents by the referring MHP. The process involved contacting the coordinator, a meeting with the parents and adolescents, completing an enrollment questionnaire, and commencing program participation. All the interviewed referring MHP also expressed satisfaction with the program’s referral process (see quote 2 in Online Supplements). Therefore, it is recommended to establish a quick and flexible single-entry point, open to all referrals, including self-referrals by the adolescents themselves [20, 21, 52]. This is consistent with many programs that adopt a self-referral policy [46] and with the personal recovery approach [20, 52, 53].

**Online presence and social media engagement.** It is recommended to enhance program visibility through the use of digital media, including an appealing and dynamic website tailored for youth associated with an active presence across social media platforms (e.g., Instagram, Facebook). The online platforms should provide comprehensive information on services, program activities, and community events. A transparent, clear, and detailed online representation can increase adolescent engagement and extend outreach to a broader audience, thus avoiding the need for intermediaries, facilitating direct communication with adolescents (including non-clinical peers), and ensuring an interface that resonates with their language. All the reviewed programs maintain an active website and most also have a social media presence [46]. An online presence can foster user empowerment by providing a space for adolescents to share their personal narratives, provide peer support, and serve as a medium for public health stigma reduction initiatives [46].

**Adolescent-oriented facilities and youth centers**. It is recommended to make users feel welcome and safe by operating adolescent-friendly facilities with a non-clinical appearance, content and environment to provide a relaxing, inviting physical space [51]. This is likely to increase adolescent engagement in these programs [52]. The coordinators emphasized the need to locate the community/youth center in a safe, secure, and accessible neighborhood that would allow adolescents to commute in the evening using public transportation. The center itself should have a permanent, pleasant and inviting office for meetings with adolescents and parents, as well as a space suitable for group activities. Youth centers (drop-in/hubs) should be colorful, fun, appealing, set up to host leisure activities and social events, equipped with the necessary resources, e.g., a kitchen, washing machine, computers, etc. A youth club is also a good way to encourage proactive activity by offering games (such as table tennis, billiards, bowling, board games, etc.), and social interactions (such as watching movies, pizza nights, etc.). Finally, proximity to a soccer/basketball field and/or a skate park may be an advantage.

**Program content**. It is recommended that programs incorporate arts, leisure activities and potential for employment (for adolescents aged 16 and over). This will contribute to the strengthening of a positive identity and the personal recovery process of these adolescents [47]. Engaging in these activities fosters social integration with peer groups and the community at large (in case of employment), strengthens relatedness, alleviates boredom and redirects focus from the MHC and its associated social difficulties. This can enable adolescents to establish routines centered on proactive actions and strengths [47], consistent with the personal recovery approach [17].

Adolescents reported that engaging in the arts elicited feelings of joy, flow, pleasure, release, pride, and a sense of meaning, thus contributing to a general sense of talent cultivation [47]. This is consistent with findings on adults with MHC [54], as well as findings pointing to the contribution of arts to better mental health and resilience [55], and that of leisure activities to a sense of empowerment, meaning, and relatedness [56]. Almost half of the programs presented in our scoping review primarily offered leisure activities [46].

*Structured*,* consistent*,* and continuous social activities to support reintegration.* Given the profound psychosocial benefits of engagement with the arts, leisure activities and employment, and in line with many stakeholders’ recommendations of “structured leisure time” for adolescents [47], it is recommended that service providers prioritize structured leisure group activities, which can serve as an ideal platform for fulfilling adolescents’ needs for competence, relatedness and autonomy [47]. By encompassing diverse forms of artistic expression (art making, performing arts, music, sports, etc.), these activities, mediated by adults modeling appropriate social behaviors, aim to enhance social skills and interactions [47]. Workshops focused on enjoyment and stress relief can further empower adolescents and enhance their well-being [46, 47]. Moreover, encouraging adolescents to select their activities enhances a sense of autonomy, involvement and subjective well-being [57].

To support reintegration, it is recommended that coordinators prepare adolescents for upcoming social interactions through the provision of comprehensive information to address their concerns and reduce anxiety. This involves helping them align expectations, which can be done by outlining the anticipated aspects of the meeting, general rules, guidelines, the meeting agenda, group composition, and behavioral expectations. Another way to support reintegration is by including mentors in joint leisure activities, who can provide opportunities for positive modeling and the creation of peer interaction through the mentors’ mediation. This also addresses their report on the lack of meaningful social activities [47].

*Creating a closed intimate group for adolescents.* It is recommended to establish closed intimate groups, facilitated by the coordinators with a professional facilitator (youth guide), consisting of a maximum of eight participants who share common interests. These groups should meet weekly and engage in meaningful activities involving active participation (e.g., sports, dancing, martial arts, etc.) or creative endeavors (e.g., carpentry, cooking, art, music, sculpture, writing, etc.). Playing introductory games during initial meetings can foster bonding, discover common interests, similarities, and facilitate integration. Once the group achieves cohesion and solidarity, group members can discuss opening the group to new participants. In this case, a more flexible approach, such as an “open studio,” [58, 59] can allow adolescents to engage in individual forms of artwork without criticism or judgment. The emphasis should be on creating a close-knit group that practices social and communication skills and can subsequently enable the participants to initiate and organize social meetings independently, without the involvement of adults.

**Mentoring and peer-support**. In the specific community-based program, the mentors, who are volunteer youth supporters, act as “social crutches.” They help the adolescents navigate social interactions by imparting social skills through modeling. As shown in the findings, while the adolescents and their parents viewed the authentic connection with mentors as the essence of mentoring, the steering committee members perceived the mentors as facilitators of social integration and active engagement, who helped expand the adolescents’ social networks and promoted active participation beyond mere companionship. This points to a perception gap between the program’s designers and consumers, and emphasizes the need for consistent role definitions for mentors [47]. Therefore, we propose that the role definition of mentors considers the needs and level of independence unique to each accompanied adolescent.

As shown in our scoping review, 30% of the programs offer peer support [46]. Incorporating peer support into programs for adolescents with MHC can be beneficial for adolescents, by enabling them to connect with peers close in age with their own experiences of MHC, and obtain valuable assistance and guidance [60–62]. Hence, an increasing number of programs have adopted peer support initiatives in recent years [60, 62]. Although there are few studies on the effectiveness of peer support in adolescents’ mental health services, research on adults indicates positive outcomes, such as improved engagement and shorter hospitalization stays [63, 64]. However, it is crucial to address the challenges associated with youth peer support, including the need for comprehensive training, supervision, clear role description, and considerations related to their age and the workload of the peers involved [60, 62, 64].

**Community advocacy**,** involvement and collaboration**. It is recommended that programs actively promote adolescents’ participation in stigma reduction campaigns by organizing events within the community and through media channels, as is the case for almost half of the reviewed programs [46]. Collaborating with diverse community services and local resources such as arts programs for adolescents [55] and the scouts can enhance program visibility and attract additional funding from donors, associations, and partners. Hosting community events led by and for adolescents contributes to their empowerment and awareness of mental health and stigma [46].

It is also recommended to offer adolescents with MHC opportunities to volunteer in the community. Engaging in volunteer activities within the community is a way to reinforce adolescents’ sense of belonging, competence and cultivate a spirit of contribution to the community. It can also contribute to forming groups through participating in volunteer opportunities as a group, in different forums, such as close intimate peer groups for adolescents with MHC, adolescents and their mentors, adolescents and their parents, etc.

Finally, it is recommended that programs provide their graduates with continuity of care and a smooth transition by collaborating with other community programs intended for young adults (aged 18 and older). This can encourage further cultivation of talent and the strengthening of a positive identity.

**Research and evaluation**. Transparency must be guaranteed to reduce potential research biases by employing an external, disinterested research team to evaluate consumer satisfaction [20, 65]. It is necessary to include consumers (adolescents) as participants in these studies and to encourage them to articulate their opinions and needs regarding the service [21, 24, 66]. Although most programs reviewed claimed to have an internal research team involving consumers [46], there are few publications of these studies and mostly unavailable online. Inconsistencies in terminology exist among researchers, both in defining the population (e.g., youth with mental illness, serious mental disorder, MHC/difficulties/issues, etc.) and defining the service. Standardizing terminology would facilitate continuity of care, inter-service collaborations, address the treatment gap, enhance service accessibility, strengthen the literature, and promote a non-stigmatized discourse on mental health.

## Limitations

While the CCR model offers a valuable conceptual framework, several limitations should be acknowledged. Primarily, the model has not yet been empirically tested, which necessitates further research to validate it. Given that the model is grounded in a specific context, its generalizability to diverse cultural, economic, and systemic environments is a key consideration, particularly where resources and policy environments may differ. Consequently, future studies are needed to examine the feasibility and effectiveness of the model in a variety of settings to strengthen its practical and policy relevance.

## Conclusions

Care for adolescents’ mental health should be a top priority for policymakers, especially considering the early onset of MHC, which is prevalent and expected to increase [7, 8]. Policymakers play a vital role by funding programs for adolescents with MHC, reducing access barriers, recruiting MHP, providing training and supervision for staff, and offering suitable structured social and leisure activities. As depicted in the CCR model, service providers should create supportive, structured environments empowering adolescents with MHC to enhance competence, autonomy, and social connections independently, while acknowledging the social, emotional, and functional aspects of adolescents’ personal recovery process. Continuity of care among all care providers is essential to optimize the personal recovery of adolescents while emphasizing the adolescents’ agency, encouraging them to engage actively in decision-making, goal setting, and determining their path to reintegration into the community and personal recovery. The CCR model offers a valuable framework for informing adolescent mental health policy, emphasizing early intervention in the community, continuity of care, and practical strategies to empower adolescents and prevent MHC escalation.

## Supplementary Information

Supplementary Material 1

## Data Availability

Data is provided within the manuscript or supplementary information files.

## Abbreviations

ADHD: attention deficit hyperactivity disorder
CCR: continuity of community reintegration
MHC: mental health conditions
MHP: mental health professionals
