# Supplementary material for: Personal recovery of adolescents with mental health conditions in the community: empirically-based practical implications
Source: Isr J Health Policy Res. 2025 Oct 30;14:62. doi: 10.1186/s13584-025-00725-0 (PMC12573885; doi:10.1186/s13584-025-00725-0)
Supplement: Supplementary file 1 — Supplementary Material 1 [file 13584_2025_725_MOESM1_ESM.docx]

**Personal recovery of adolescents with mental health conditions in the community:**

**Empirically-based practical implications**

**Supplementary Material**

1. In the words of a committee representative from *Amitim for Youth*:

It is impossible, in the case of adolescents, to talk about rehabilitation, because there is no long-term history of coping [with MHC] that necessitates rehabilitation... In general, some people coping [with MHC] are disturbed by the term ‘rehabilitation’... it is not exactly rehabilitation or treatment. So... ‘community integration’ is the correct term here.

1. All of the interviewed referring MHP expressed satisfaction with the program’s referral process. As MHP 2 stated:

It [the referral process] works well. I don’t have any changes to suggest. I coordinate with the pupils who I think [are suitable for the program] and make the referral... [I send the coordinator] the referral form, we talk about the child a little, she invites the parents and child to come for a preliminary meeting to match the child with a mentor. I would not change anything... in either the identification process or the referral process.
